# Kupffer cells promote T-cell hepatitis by producing CXCL10 and limiting liver sinusoidal endothelial cell permeability

**DOI:** 10.7150/thno.44960

**Published:** 2020-06-01

**Authors:** Shen Dai, Fengming Liu, Zhongnan Qin, Jinyan Zhang, Jiayi Chen, Wen-Xing Ding, Dechun Feng, Yong Ji, Xuebin Qin

**Affiliations:** 1 Division of Comparative Pathology, Tulane National Primate Research Center, Covington, LA 70433, USA.; 2 Department of Neuroscience, Temple University Lewis Katz School of Medicine, Philadelphia, PA 19140, USA.; 3 Key Laboratory of Cardiovascular and Cerebrovascular Medicine, Key Laboratory of Targeted Intervention of Cardiovascular Disease, Collaborative Innovation Center for Cardiovascular Disease Translational Medicine, Nanjing Medical University, Nanjing, Jiangsu 211166, China.; 4 Department of Immunology and Microbiology, Tulane University School of Medicine, New Orleans, LA 70112, USA.; 5 Department of Hepatobiliary and pancreatic surgery, Shanghai General Hospital, Shanghai Jiao Tong University School of Medicine, Shanghai 200080, China.; 6 Department of Clinical Laboratory Department, Shanghai Tenth People's Hospital of Tongji University, Shanghai 200072, China.; 7 Department of Pharmacology, Toxicology and Therapeutics, The University of Kansas Medical Center, Kansas City, Kansas 66160, USA; 8Laboratory of Liver Diseases, National Institute on Alcohol Abuse and Alcoholism, National Institutes of Health, Bethesda, MD 20892, USA.; 8 Laboratory of Liver Diseases, National Institute on Alcohol Abuse and Alcoholism, National Institutes of Health, Bethesda, MD 20892, USA.

**Keywords:** Hepatitis, Kupffer cells, CXCL10, Sinusoid endothelial cells, Cell ablation

## Abstract

**Rationale:** Kupffer cells (KCs) play a crucial role in liver immune homeostasis through interacting with other immune cells and liver sinusoidal endothelial cells (LSECs). However, how KCs exactly interact with these cells for maintaining the homeostasis still require the further investigation. CXCL10 is a chemokine that has been implicated in chemoattraction of monocytes, T cells, NK cells, and dendritic cells, and promotion of T cell adhesion to endothelial cells. Although CXCL10 is also known to participate in the pathogenesis of hepatic inflammation, the degree to which it is functionally involved in the crosstalk between immune cells and regulation of immune response is still unclear.

**Methods:** To dynamically investigate the function of KCs, we used our recently developed rapid cell ablation model, intermedilysin (ILY)/human CD59 (hCD59)-mediated cell ablation tool, to selectively ablate KC pool under normal condition or concanavalin A (Con A)- induced hepatitis. At certain time points after KCs ablation, we performed flow cytometry to monitor the amount of hepatic infiltrating immune cells. mRNA array was used to detect the change of hepatic cytokines and chemokines levels. Cytokines and chemokines in the serum were further measured by LEGENDplex^TM^ mouse proinflammatory chemokine panel and inflammation panel. Evans blue staining and transmission electron microscopy were used to investigate the interaction between KCs and LSECs in steady condition. CXCL10 neutralizing antibody and CXCL10 deficient mouse were used to study the role of CXCL10 in immune cell migration and pathogenesis of Con A-induced hepatitis.

**Results:** At steady state, elimination of KCs results in a reduction of hepatic infiltrating monocytes, T, B, and NK cells and a list of cytokines and chemokines at transcriptional level. In the meantime, the depletion of KCs resulted in increased sinusoidal vascular permeability. In the pathological condition, the KCs elimination rescues Con A-induced acute hepatitis through suppressing proinflammatory immune responses by down-regulation of hepatitis-associated cytokines/chemokines in serum such as CXCL10, and recruitment of infiltrating immune cells (monocytes, T, B, and NK cells). We further documented that deficiency or blockade of CXCL10 attenuated the development of Con A-induced hepatitis associated with reduction of the infiltrating monocytes, especially inflammatory Ly6C^hi^ monocytes.

**Conclusions:** This study supports the notion that KCs actively interact with immune cells and LSECs for maintaining immune response and liver homeostasis. Our data indicate that the interplay between KCs and infiltrated monocytes via CXCL10 contribute to Con A-induced hepatitis.

## Introduction

The liver is essential for metabolic activities, nutrient storage and detoxification. It also serves as a critical surveillance system for gut-derived pathogens and maintains immune homeostasis by producing several key immune components (complement, acute phase, and coagulation) to participate in immune reactions or secrete immune regulatory chemokines and cytokines to suppress the immune response 1,2. The liver immune homeostasis and surveillance are largely regulated within the hepatic sinusoid, in which Kupffer cells (KCs) are located as part of the liver reticuloendothelial system (also known as the mononuclear phagocyte system) 3,4. KCs, the residential liver macrophages, are the largest population of tissue macrophages 4,5. KCs constitute approximately 10% of hepatic cells and comprise nearly one-third of non-parenchymal cells in the liver 6. The critical role of KCs and infiltrating monocytes/macrophages in maintaining immune homeostasis of the liver and in the pathogenesis of liver injury has been established 7; however, the interaction of KCs with immune and non-immune cells in the immune tolerance and inflammatory responses requires further investigation. Anatomically, KCs line on top of the endothelium in the hepatic sinusoids and interact with hepatocytes through the sinusoid endothelium 1. Liver sinusoidal endothelial cells (LSECs) and KCs, serve as physical platforms for recruitment and anchoring of blood-borne immune cells in the liver 5. The interplay of KCs and LSECs has been suggested to play an important role in immune tolerance and inflammatory responses in the liver 5. However, whether this interplay participates in maintaining vascular integrity remains elusive and requires further investigation.

In the hepatic sinusoids, KCs are in close contact with infiltrating monocytes, T cells, B cells, and liver-resident immune cells such as NK cells and NKT cells 5. In the steady state, the autoregulatory downregulation of IL-10 expression in KCs has been documented to account for important down-regulatory steps for humoral immune tolerance in the liver sinusoid 8. In the pathological state, KCs may activate infiltrating immune cells including monocytes, T cells, and NKT cells, leading to immune responses against bacterial infection and causing liver immunopathology 9,10. Dynamic interactions between the numerous immune cell populations in the liver are essential for maintaining immune balance and overall tissue health 11. These interactions have also been suggested to play a central role in the pathogenesis of liver injury, making them an attractive therapeutic target 7,12. However, how KCs exactly interplay with other immune cells such as monocytes, T cells, B cells, NK cells, and NKT cells in normal and pathological conditions remains unclear. The molecular mechanisms underlying KC-mediated immune tolerance and activation are largely unknown.

KCs are major source of many kinds of cytokines and chemokines 7. Chemokines and their corresponding receptors play a crucial role in orchestrating the influx of immune cells into injured or disease organs, driving inflammatory responses 13. They serve as important messengers between hepatic local cells and circulating immune cells. Among the various chemokine-receptor axes, the CXCL9-11/CXCR3 axis is unique. This axis has three ligands (CXCL9, CXCL10 and CXCL11) that bind to CXCR3 and alternatively regulate downstream events 14. CXCR3 is highly expressed in infiltrating lymphocytes. Mice with a genetic deletion of CXCR3 are more prone to liver injury, which is mediated by the loss of antifibrogenic and angiostatic properties of CXCL9 on hepatic stellate cells (HSCs) and LSECs 15,16. CXCL11/CXCR3 binding induces an immune-tolerant state that is characterized by anti-inflammatory Th2 polarization 17,18. Deletion of CXCL10 inhibits murine liver fibrosis 19, and the neutralization of CXCL10 ameliorates experimentally induced liver injury in vivo 20,21. Furthermore, serum and intrahepatic CXCL10 levels in patients are positively associated with the severity of hepatitis C virus (HCV)-induced liver disease 15,22. Although KCs and certain chemokines are known to be implicated in development of hepatic inflammation, the functional role of chemokines such as CXCL10 produced by KCs in mediating the interaction with the infiltrated immune cells and the immune response is still obscure.

To address these questions, we need to utilize the in vivo depletion approach to eliminate the KC population for loss-of-function studies. Currently, common models employed for eliminating KCs or blocking KC functions are clodronate liposomes, chloride or diphtheria toxin/diphtheria toxin receptor (DT/DTR), and gadolinium 3,23,24. However, these approaches induce slow cell death (days) by initiating apoptosis and/or inhibition of KC function 25,26, which makes it difficult to clearly differentiate KC-associated immune and non-immune responses from KC loss to regeneration phases. These limitations prevent us from comprehensively and systemically studying the molecular and cellular responses of these sinusoid cells after eliminating KCs in normal and pathological conditions. In this paper, we applied our recently generated Cre induced-human CD59 (hCD59) transgenic line (ihCD59), a model that can rapidly and specifically ablate a specific cell population by injection of intermedilysin (ILY) for selectively ablating KC pools 27-30. Upon acute elimination, we dynamically examined the hepatic infiltrating immune cell changes via flow cytometry, the vascular integrity via Evan blue staining, and the cytokine and chemokine changes via mRNA and protein tissue array analysis. We demonstrate that the acute elimination of KCs down-regulates immune interactions in both steady and pathological conditions and increases vascular permeability. Our data also indicates that the interplay between KCs and monocytes via CXCL10 contributes to Con A-induced hepatitis.

## Methods

### Mice

Animal studies were approved by the Institutional Animal Care and Use Committees at Tulane University and Temple University. *LysM-Cre^+^* (JAX 004781) and *CXCL10^-/-^*(006087) mice were purchased from the Jackson Laboratory and housed in animal facilities at both institutions. The *ihCD59^+/-^* mice were previously generated and backcrossed with C57BL/6 background for at least eight generations 27. All other mice used in this study were on C57BL/6 background. *LysM-Cre^+^* mice were crossed with *ihCD59^+^* mice to generate *LysM-Cre^+^ihCD59^+^* double transgenic mice.

### ILY purification

His-tagged recombinant ILY was purified as described previously 27,28. Endotoxin was removed using an Endotoxin Removal Kit (Pierce, Rockford, IL). The concentration and purity of ILY were determined by SDS-PAGE. The activity of ILY was determined by in vitro hemolytic assay described in previous work 28. The IC50 of ILY used in current study is 58.6 pM. In some experiments, ILY was boiled for 5 min to generate heat-inactive ILY (hi-ILY).

### Patients' liver tissue specimen

Patients who underwent orthotopic liver transplantation in 2019 were enrolled in the study. Liver tissue was obtained from patients who underwent orthotopic liver transplantation donation after cardiac death (DCD) in 2019 in Shanghai General Hospital Affiliated to Shanghai Jiao Tong University. HCV diagnostic criteria from to the Chinese Diagnostic Criteria for Chronic Hepatitis B (2015 edition) were used.

The study was approved by the Ethics Committee of Shanghai General Hospital Affiliated to Shanghai Jiao Tong University. The methods were carried out in accordance with the Declaration of Helsinki and its later amendments or comparable ethical standards. Liver grafts were obtained from DCD. No donor livers were harvested from executed prisoners. The participants or the next of kin gave their informed consent for the study.

### Macrophages depletion by clodronate liposomes

Clodronate liposomes and control liposomes (PBS) were purchased from Liposoma BV (Amsterdam, The Netherlands) and stored at 4 °C. To deplete macrophages in vivo, mice received 10 μl/g body weight of clodronate liposomes or control liposomes (PBS) by i.v. injection.

### Con A-induced acute liver injury and ILY treatment

Mice received either 1 ILY (150 ng/g, i.p.) or 3 ILY injections (100 ng/g, i.p., 2 h intervals). 24 h after the first ILY injection, Con A was administered at a dose of 12 mg/kg by i.v. injection. Serum was obtained by tail bleeding at different time points after Con A injection for further studies. 24 h after Con A injection, liver tissues were fixed in 4% paraformaldehyde (PFA) for histological analysis, and serum was collected for ALT/AST measurement.

### Liver fixation, sectioning and immunohistochemistry

Mice were perfused with PBS. Livers were dissected out and fixed in 4% PFA overnight. Fixed liver samples were processed and embedded in paraffin. Sections of 4 μm thickness were stained with Hematoxylin and Eosin (H&E) for histological analysis.

### Immunofluorescence staining

Livers were fixed in 4% PFA overnight, transferred in 30% sucrose for two days and embedded in O.C.T compound. Cryosections of 7 μm were made and stained with rabbit anti-F4/80 (AbD Serotec, Oxford, United Kingdom), CD68 antibody and Rabbit Anti-IP10 antibody (CST, Inc, Mount Carmel, IL) separately at 4 °C overnight. After washing with PBS, the slides were stained with Alexa 594-conjugated goat-anti-rabbit antibody (Invitrogen, Carlsbad, CA) for 1 h and mounted with Fluoroshield with DAPI (Sigma, St. Louis, MO). The images were visualized by using LEICA TCS SP8 Confocal microscope (Leica Microsystems Inc, Wetzlar, Germany).

### Isolation of the infiltrating immune cells in liver

Mice were euthanized by CO_2_ and perfused with ice-cold PBS from left heart ventricle. Livers were harvested and mashed with plungers of 5 mL syringe in PBS and passed through 70 μm cell strainers. To enrich hematopoietic cells, the suspension was further processed by Percoll gradient centrifugation. Briefly, the pellet was re-suspended with 10 mL 40% Percoll and carefully layered over 3 mL 70% Percoll prior to density centrifugation (500xg 30 min with no brake). After centrifugation, the layer at the junction of 40/70% Percoll was collected and washed twice with 10 mL PBS (500xg, 8 min). Cell pellet was further washed in flow cytometry staining buffer (PBS with 2% BSA) and re-suspended in 100 μl staining buffer and processed for staining.

### Portal vein perfusion and KC isolation

KCs were isolated via in situ collagenase perfusion from portal vein as previously described in 31. Briefly, mice were anesthetized with avertin (200 mg/kg, i.p.) and the portal vein was cannulated under aseptic conditions. The liver was perfused with warm EGTA solution [5.4 KCl, 0.44 KH_2_PO_4_, 140 NaCl, 0.34 Na_2_HPO_4_, 0.5 EGTA, and 25 tricine (pH 7.2), all in mmol/L] for 10 min and followed by pre-warmed digestion buffer (0.075% collagenase IV in HBBS containing 1 mM CaCl2) for 10 min at a speed of 3 mL/min. After perfusion, the liver was carefully dissected out and digested in digestion buffer at 37 °C for 30 min, then mashed and passed through a 70 μm cell strainer. Cell suspension was centrifuged at a speed of 500 g for 4 min at 4 °C. The pellet was re-suspended in 8 mL 40% Percoll and centrifuged for 30 min at 2000 rpm at 4 °C. The pellet containing KCs and endothelial cells was kept and washed in flow cytometry staining buffer (PBS with 2% BSA) and resuspended in 100 μl staining buffer and processed for staining.

### Flow cytometry staining and acquisition

Single cell suspensions were incubated with 1:200 anti-CD16/32 (FcγRIII/II) antibody for 15 min to block non-specific Fc receptor binding. The antibody cocktails were added and incubated at 4°C for 30 min. The antibodies used are listed in Table S**1**. DAPI (Invitrogen) and SPHERO AccuCount Particles (Spherotech Inc, Lake Forest, IL) were added before acquisition to distinguish live/dead cells and access absolute cell numbers, respectively. Gating strategy of each cell population is shown in Figure S8. Data was acquired on a LSRII flow cytometer (BD, San Jose, CA). All data were analyzed using FlowJo 10.6.1 (Tree Star Inc. Ashland, Oregon).

### Biochemical analysis

Serum ALT/AST levels were determined using an IDEXX chemistry analyzer system (IDEXX Laboratories, Westbrook, ME).

### mRNA array

RT² Profiler™ PCR Array Mouse Cytokines & Chemokines (PAMM-150Z, Qiagen, Hilden, Germany) was used to profile the expression of 84 cytokines and chemokines in mouse livers. Total RNA was extracted from liver using RNeasy Mini Kit (Qiagen) and the concentration was quantified using a nanodrop. Total RNA (500 ng) was converted to cDNA using RT^2^ First Strand Kit (Qiagen) according to the manufacturer's protocol. cDNA synthesis solution was further diluted with water and served as the template of the RT^2^ Profiler PCR Array Kit to determine the expression of 84 genes including major cytokines/chemokines and 5 housekeeping genes. The assay was performed in Roche Lightcycler 96 system following the kit's protocol. The data was loaded and analyzed using web-based software.

### Cytokine/chemokine measurement

Cytokines (IL-1α, IL-1β, IL-6, IL-10, IL-12p70, IL-17A, IL-23, IL-27, MCP-1, IFN-β, IFN-γ, TNF-α, and GM-CSF) and chemokines (CCL2, CCL5, CXCL10, CCL11, CCL17, CCL3, CCL4, CXCL9, CCL20, CXCL5, CXCL1, CXCL13, and CCL22) in the serum were measured by LEGENDplex^Tm^ mouse proinflammatory chemokine panel and LEGENDplex^Tm^ mouse inflammation panel (Biolegend, San Diego, CA) following the manufacturer's protocol and acquired via LSRII flow cytometry. In some experiments, serum CXCL13 level was measured using a mouse CXCL13/BLC/BCA-1 DuoSet Elisa Kit (R&D DY470, Minneapolis, MN).

### CXCL10 neutralization

To block the effect of CXCL10, mice were intraperitoneally injected with 100 μg CXCL10 neutralizing antibody (MAB466, R&D), rat IgG2A isotype control antibody (MAB006, R&D) or PBS 2 h before Con A injection.

### Evans blue assay

To determine the vascular permeability, mice were intraperitoneally injected with Evans Blue solution in PBS (50 μg/g body weight, i.v.) 30 min before anesthesia and portal vein perfusion. Mice were perfused with ice-cold PBS (3 mL/min, 10 min) from portal vein to remove the dye in circulating system, especially sinusoid of liver. Different tissues were collected and weighed. Evans blue infiltrated in tissues was extracted by incubating in formamide (1 mL/g tissue) at room temperature for 48 h. The absorbance of the supernatant was measured at 620 nm. The concentration of Evans blue in samples was calculated using a standard curve of Evans blue.

### Transmission electron microscopy

Livers were perfused and fixed with 2.5% glutaraldehyde in 0.1 mol/L phosphate buffer (pH 7.4), followed by 1% OsO4. After dehydration, thin sections were stained with uranyl acetate and lead citrate. Images were taken digitally under a JEM 1011CX electron microscope (JEOL, Peabody, MA) as previously described in 32.

### Statistics

Data are expressed as mean ± SEM. To compare values obtained from multiple groups over time, two-way analysis of variance (ANOVA) was used, followed by Bonferroni post hoc test. To compare values obtained from two groups, the unpaired Student's *t*-test was performed. Statistical significance was taken at the P < 0.05 level.

## Results

### Establishment of ILY/hCD59-mediated KC ablation model

**Figure 1:**
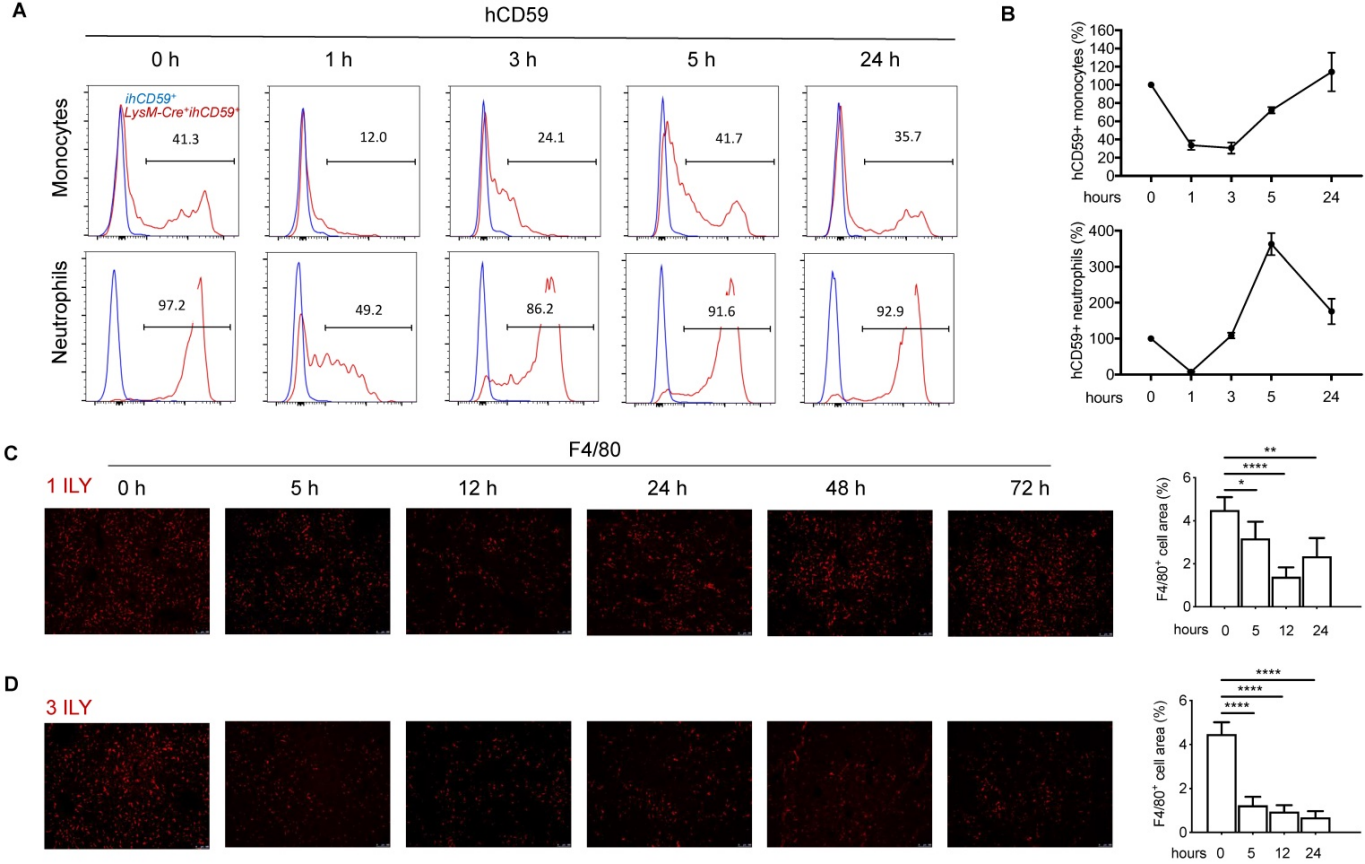
** Dynamic changes of the circulating myeloid cells and Kupffer cells (KCs) in *LysM-Cre^+^ihCD59^+^* mice injected with ILY of two dosing regimens. (A)** Histogram of hCD59 expression on circulating monocytes (upper panel) and neutrophils (lower panel) at 0 h (baseline before ILY injection), 1, 3, 5, and 24 h after a single intraperitoneal injection (i.p.) of 150ng/g ILY in ihCD59+ (blue line) or *LysM-Cre+ihCD59^+^* mice (red line). **(B)** Dynamic changes of hCD59-positive (ihCD59+) circulating monocytes (upper) and neutrophils (lower) after the single ILY injection in *LysM-Cre+ihCD59^+^* mice. The number of hCD59+ cells before ILY injection (or at 0 h) was set as 100%. **(C, D)** Immunofluorescence staining (left) and quantification (right) of F4/80 positive (F4/80^+^) KCs in the liver of *LysM-Cre+ihCD59^+^* mice (n = 3 for each time point). Mice were treated with either 1 ILY (C, 150 ng/g, i.p.), or 3 ILY injections (D, 100 ng/g, i.p. at 2 h intervals). Data are shown as Mean ± SEM. * P<0.05; ** P<0.01; **** P<0.0001, as determined by unpaired two-tailed Student's t-test.

To investigate the interplay of KCs with infiltrating immune cells in the liver, we utilized our recently developed ILY/ihCD59-mediated cell ablation tool to specifically deplete KCs. First, we crossed Cre-inducible hCD59 transgenic mice (*ihCD59^+^*) with myeloid cell specific *LysM-Cre^+^* mice to generate *Lysm-Cre^+^ihCD59^+^* mice, in which hCD59 is specifically expressed on myeloid cells in blood (~40% of monocytes and 94.3% of neutrophils) (Supplementary Figure S1A) and KCs in liver (Supplementary Figure S1B), but not on NK cells, T cells, and B cells in blood (Supplementary Figure S1A) or sinusoidal endothelial cells (LSEC) and NKT cells in liver (Supplementary Figure S1B). The ratio and number of hCD59^+^ circulating monocytes and neutrophils significantly decreased at 1 h and started to recover at 3 h or 1 h respectively, then returned to normal levels at 24 h after one ILY injection (150 ng/g, i.p.) (Figure 1A and 1B). One dose of ILY also effectively depleted F4/80^+^ liver KCs in *LysM-Cre^+^ihCD59^+^*, but not *ihCD59^+^* mice (Figure 1C). Immunostaining revealed that the number of F4/80^+^ cells was significantly reduced at 5 h post injection, reached the lowest level (around 60% reduction) at 12 h and started to recover at 24 h (Figure 1C). Importantly, neither addition of ILY to the cells isolated from *LysM-Cre^+^ihCD59^+^* liver in vitro nor injection of ILY to the *LysM-Cre^+^ihCD59^+^* mice damaged other immune cells such as NK, NKT, T, and B cells (Supplementary Figure S2A and S2B).

To establish a selective and sustained KC ablation model for studying the interaction between KCs and hepatic infiltrating immune cells, we further optimized the ILY dose regimen and selected the optimal intervals for studying the immune responses after KCs ablation. As shown in Figure 1D, we documented that 3 consecutive doses of ILY (100 ng/g, i.p.) at 2 h intervals is an optimal dose regimen to achieve depletion of more than 80% of KCs, and remained at this level until 72 h after the initial ILY injection in* LysM-Cre^+^ihCD59^+^* mice (Figure 1D). To differentiate the specific effect of KC ablation from circulating monocytes ablation on hepatic infiltrating immune cells, we also analyzed the dynamic change of circulating monocytes after the 3 ILY injections (Figure S3). We found that circulating monocytes returned to baseline level at 24 h post initial ILY injection (Figure S3), while KCs remained at the lowest level at this time (24 h) after ILY injection in the same experiments (Figure 1D). Therefore, we choose 24 h after 3 ILY injections as the best time point and 14 h (when circulating monocytes were undergoing regeneration (Figure S3) and KCs were significantly depleted after the ablation (Figure 1D) as a transitional time point to dynamically investigate the interplay of KC depletion on the hepatic infiltrating immune cells and the underlying immune responses. Taken together, these data indicate that we have established a selective KC depletion model via ILY/ihCD59-mediated cell ablation tool.

### KC depletion reduced hepatic infiltrating immune cell numbers and immune response in steady state

**Figure 2:**
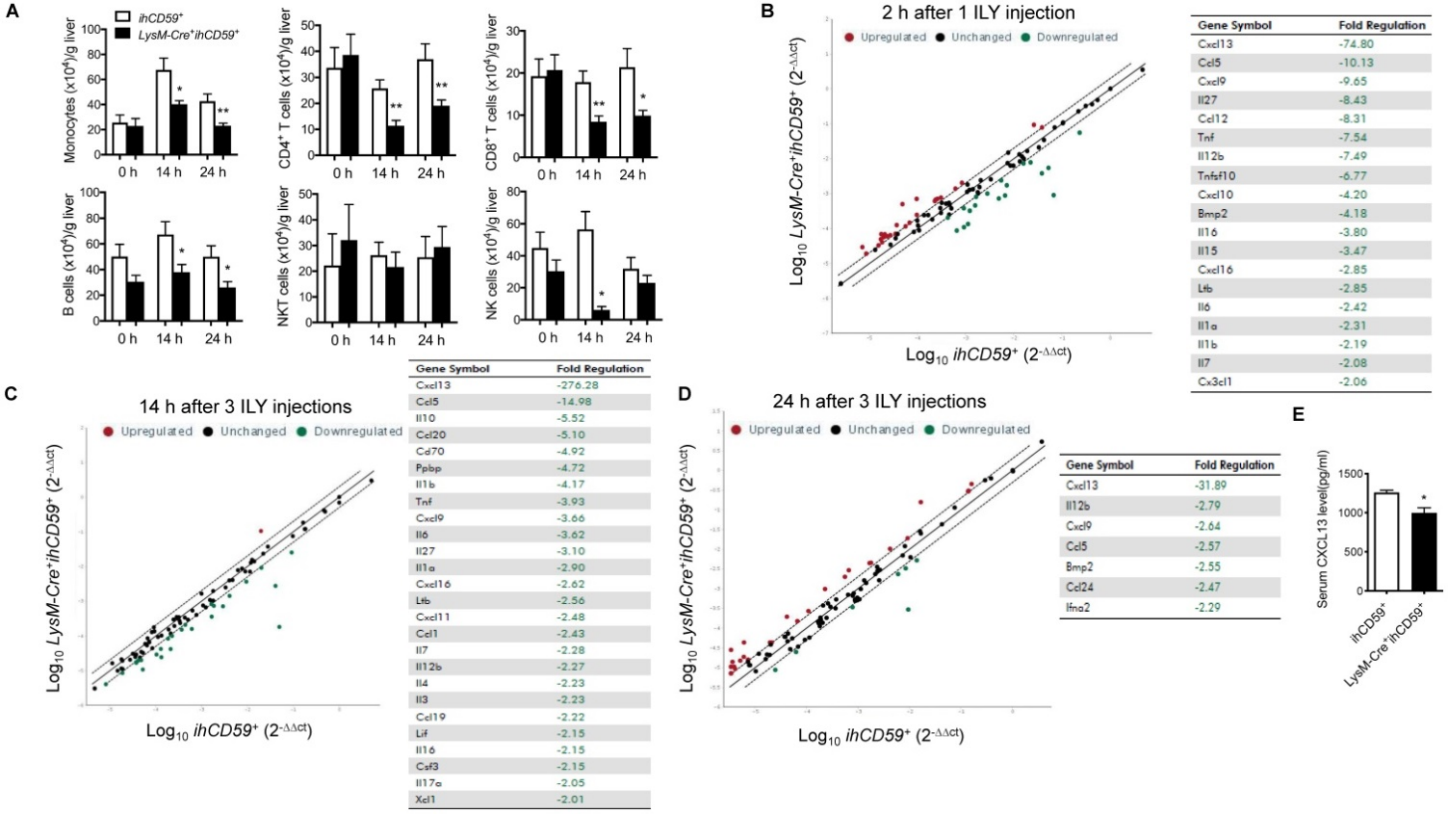
** Dynamic changes of immune cell infiltration, and cytokine & chemokine profile in the liver after the KC depletion in steady condition. (A)** Numbers of infiltrating immune cells (monocytes, T cells, B cells, NKT cells and NK cells) in livers before ILY injection (0 h) or at 14 and 24 h after 3 ILY injections (100 ng/g, i.p. at 2 h intervals) to *ihCD59^+^* and *LysM-Cre^+^ihCD59^+^* mice (n=4 at 0 h and n=6 for all other time points). **(B-D)** RT2 Profiler PCR Array analysis of the hepatic cytokine and chemokine transcripts in the *LysM-Cre^+^ihCD59^+^* and *ihCD59^+^* mice at 2 h after 1 ILY injection (B, 100 ng/g, i.p., n = 2), and at 14 h (C) and 24 h (D) after 3 ILY injections (100 ng/g, i.p. at 2 h intervals, n = 2 for each time point). Scatter plot represents the relative transcript levels for each gene obtained from *LysM-Cre^+^ihCD59^+^* mice plotted against the same gene from *ihCD59^+^* mice. Genes with fold changes >2.0 are shown in the table on the right. **(E)** ELISA analysis of CXCL13 in serum collected from *ihCD59^+^* and *LysM-Cre^+^ihCD59^+^* mice (n = 3) at 8 h after 3 ILY injections (100 ng/g, i.p. at 2 h intervals). Data are shown as mean ± SEM. * P<0.05; ** P<0.01, as determined by unpaired two-tailed Student's t-test.

We explored the interplay between KCs and hepatic infiltrated immune cells, and the underlying immune response in the steady state. After transcardial perfusion of mice, we analyzed liver infiltrating immune cells such as monocytes, T cells, B cells, NK cells and NKT cells at 14 h and 24 h after the first ILY injection of selective KC depletion model by flow cytometry (Figure 2A). We documented that 3 ILY injections significantly decreased the number of hepatic infiltrating monocytes, CD4^+^ T cells, CD8^+^ T cells and B cells except NKT cells in *LysM-Cre^+^ihCD59^+^* mice than that in *ihCD59*^+^ mice at both time points (Figure 2A). Of note, NK cells were also significantly reduced at 14 h in *LysM-Cre^+^ihCD59^+^* mice and recovered at 24 h post ILY injection (Figure 2A). Furthermore, we performed in vitro studies to investigate whether the released cellular contents of ILY-lysed cells have any toxic effect on adjacent infiltrating cells, leading to a reduction of the cell numbers observed in *LysM-Cre^+^ihCD59^+^* mice. To this end, we obtained released cellular contents of ILY-lysed cells and non ILY-lysed cells by collecting the supernatants of the *LysM-Cre^+^ihCD59^+^* hepatic cells pre-treated with saturated doses of ILY, heat-inactive ILY (hi-ILY) or PBS (Supplementary Figure S4A). The supernatants were incubated with wild type (Wt) hepatic immune cells (Supplementary Figure S4B) or splenocytes, respectively (Supplementary Figure S4C). After the incubation, we did not detect any significant differences between Wt immune cells incubated with ILY-lysed (ILY-treated *LysM-Cre^+^ihCD59^+^* hepatic cells) or non ILY-lysed cellular contents (either hi-ILY or PBS-treated *LysM-Cre^+^ihCD59^+^* hepatic cells). These results indicate that the released cellular contents of ILY-lysed cells are not harmful to other immune cells. Considering that hCD59 only expressed on monocytes (Supplementary Figure S1) and ILY does not mediate lytic effect on any hCD59 negative cells (Supplementary Figure S2A and B), our results indicate that the depletion of KCs mediates the reduction of immune cell infiltration in liver. To further confirm this notion, we utilized clodronate liposomes, another widely used monocyte and KC depletion method 30,33, to inject *Wt* mice, and demonstrated that clodronate liposomes significantly reduced number of hepatic monocytes at 12 h and 24 h. In the meantime, CD4^+^ T cells and NK cells decreased as well at 24 h after injection but not CD8^+^ T cells and B cells (Supplementary Figure S5). Compared to our model, clodronate liposome injection reduced hepatic immune cell infiltration to a lesser degree. Taken together, these results suggest that KCs participate in the maintenance of infiltrating immune cells in liver.

Cytokines and chemokines play critical roles in mediating immune cell interplays, innate and adaptive immune responses by activating and recruiting immune cells 34,35. To better understand the interaction between KCs and hepatic infiltrating immune cells, we compared transcript levels of 84 cytokine/chemokines in liver between *LysM-Cre^+^ihCD59^+^* and *ihCD59^+^* mice with ILY injections by qRT-PCR array. To track the dynamic change of these cytokine/chemokine transcripts during KC depletion, we performed analysis at 2 h post one ILY injection, 14 h and 24 h post 3 ILY injections in *LysM-Cre^+^ihCD59^+^* and *ihCD59^+^* mice. The results show that a list of cytokine/chemokine transcriptional activities were rapidly reduced in the liver of *LysM-Cre^+^ihCD59^+^* mice at 2 h (19 molecules) and 14 h (26 molecules) after ILY treatments (Figure 2B and C), and 7 molecules were still decreased at 24 h (Figure 2D). Further, most of these cytokines and chemokines decreased more at early time points (2 h and 14 h) compared to their levels at later time point (24 h). Among these cytokines/chemokines, IL-12β, Cxcl13, Cxcl9, and Ccl5 were continuously reduced at all three time points in *LysM-Cre^+^ihCD59^+^* with KC ablation compared with *ihCD59^+^* mice. We found that CXCL13, the chemokine that showed the greatest decrease in transcriptional activity after KC ablation, was also significantly reduced in serum (Figure 2E). These results indicate that rapid depletion of KCs significantly reduced the production of these cytokines and chemokines, which plays very important roles in maintaining immune homeostasis for normal liver function. In addition, we observed increased transcriptional activities of some cytokines/chemokines such as Cxcl3, Spp1, and Csf3 at 2 h (25 molecules) and 24 h (31 molecules) than 14 h (one molecule) in *LysM-Cre^+^ihCD59^+^*with ILY treatments (Supplementary Figure S6A-C). The upregulated cytokine/chemokine transcriptional activities at 2 h and at 24 h may result from acute hepatic inflammatory and regenerative responses post ILY-mediated KC ablation, respectively. Regarding the regenerative responses, we found that Spp1, also known as osteopontin, was upregulated at 14 h and 24 h in response to KC ablation, which is indicative of Spp1's role in the replenishment of KCs. This predication is also supported by the previous finding that Spp1 enhances the recruitment of macrophages and neutrophils, and triggers hepatocyte regeneration 36,37. Taken together, our results suggest that KCs play an important role in maintaining hepatic homeostasis through modulating immune cell infiltration and immune response in steady condition.

### KC depletion completely protected against concanavalin (Con A)-induced acute liver injury via reducing hepatic immune cell infiltration and suppressing immune response

**Figure 3:**
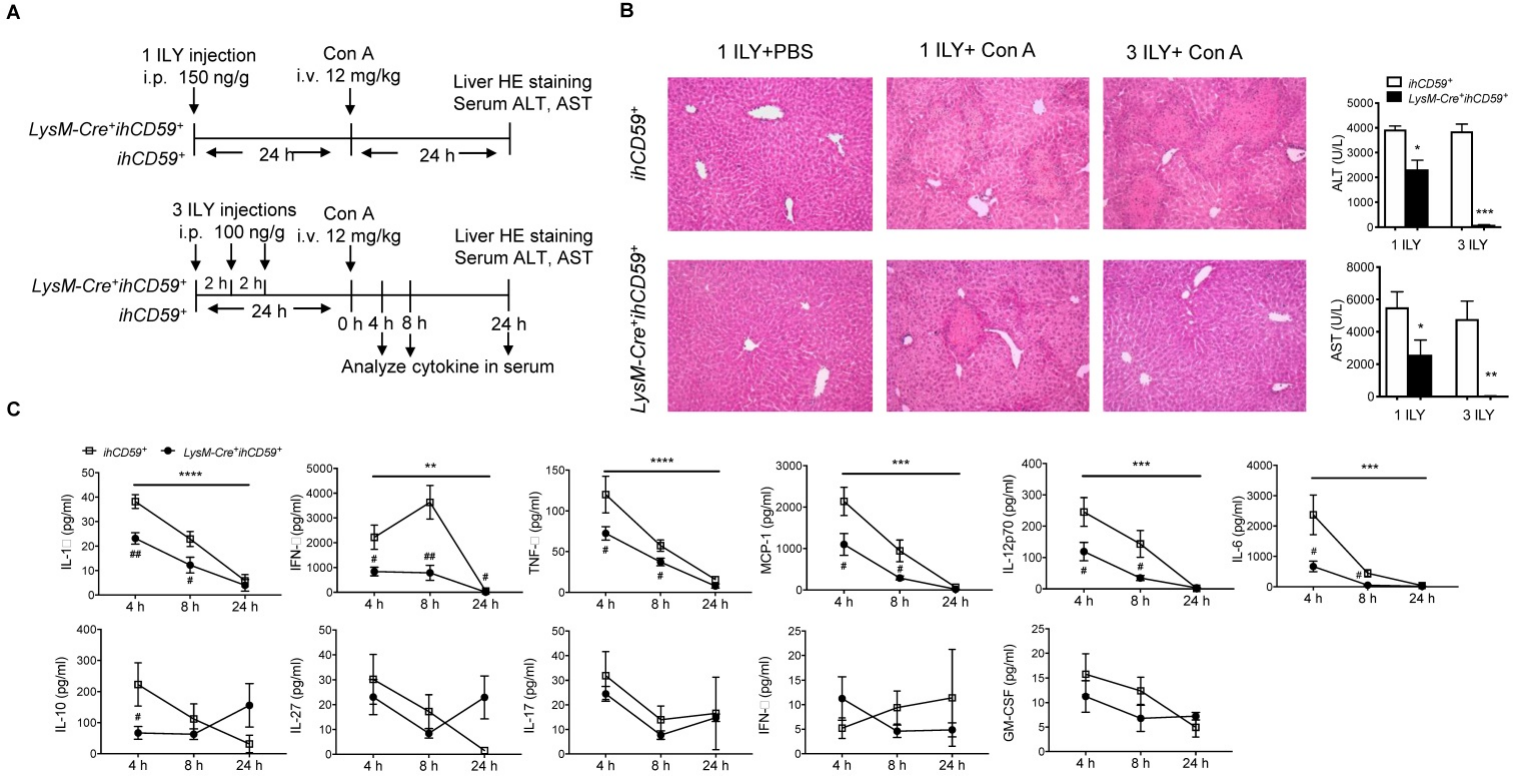
** KC depletion completely protects against concanavalin A (Con A)-induced acute liver injury associated with reduced inflammatory cytokines in serum. (A)** Scheme of experimental procedures for the mouse genotypes, and the timeline of ILY and Con A treatments and further analysis. *ihCD59^+^* and *LysM-Cre^+^ihCD59^+^* mice were treated with 1 ILY (middle, 150 ng/g, i.p.) or 3 ILY injections (right, 100 ng/g, i.p. at 2 h intervals), followed by the injection of Con A (12 mg/kg i.v.) 24 h later. Serum were collected at 4 and 8 h after Con A injection by tail bleeding. **(B)** H&E stained liver sections and serum ALT and AST of *ihCD59^+^* (n = 3) and *LysM-Cre^+^ihCD59^+^* (n = 3) at 24 h after the Con A injection. Left panel is another experimental control group treated with 1 ILY injection (200 ng/g, i.p., n = 2) only. **(C)** Serum level of cytokines at 4, 8 and 24 h after Con A treatment in *ihCD59^+^* and *LysM-Cre^+^ihCD59^+^* mice were analyzed by Legendplex flow assay (n = 6 for 4 and 8 h and n = 2 for 24 h). Data are shown as mean ± SEM. ** P<0.01; *** P<0.0005; **** P<0.0001 (two-way ANOVA) in comparison with *ihCD59^+^* group. # P<0.05; ## P<0.01 as determined by unpaired two-tailed Student's t-test.

**Figure 4:**
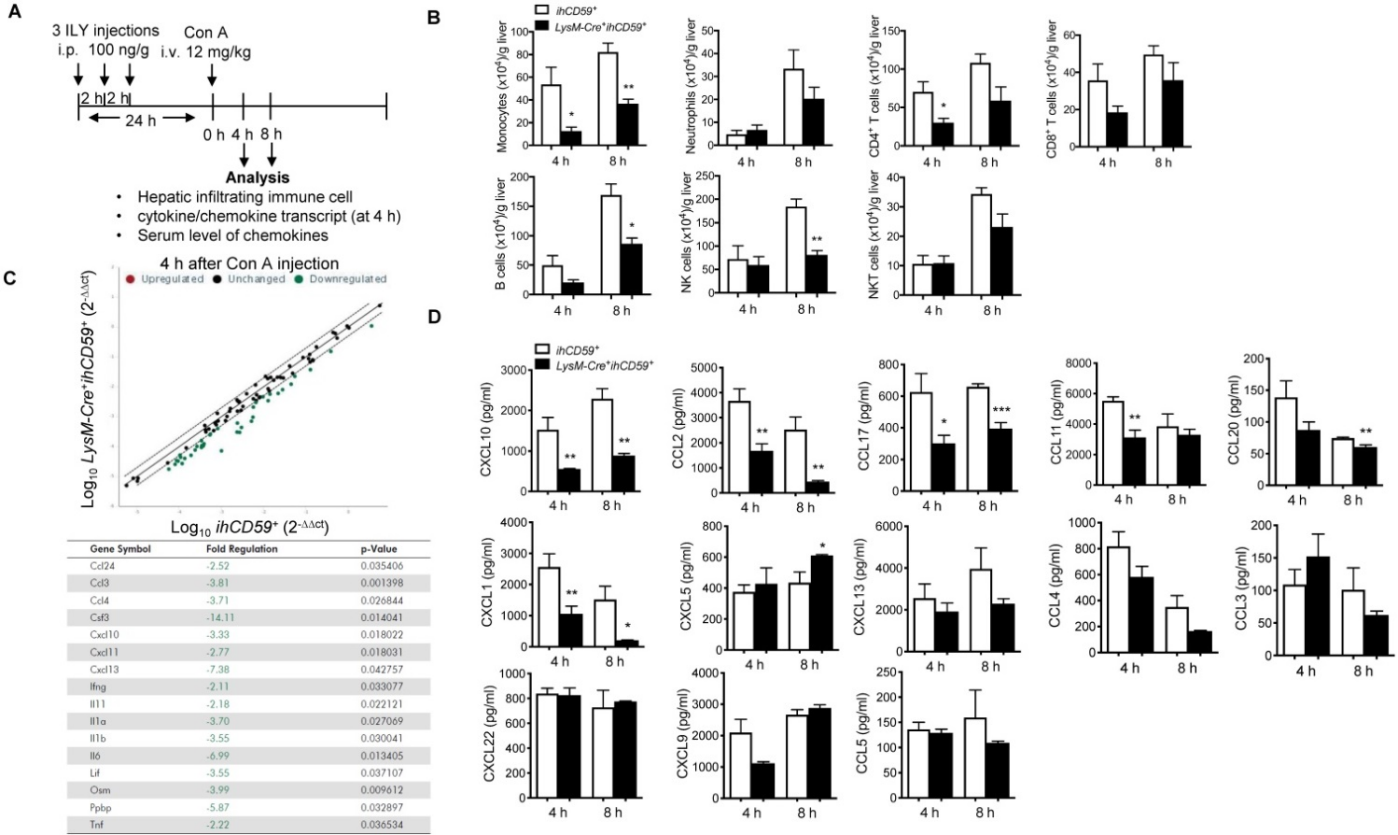
** Immune cell count and chemokine profile in the liver with Con A-induced acute injury after selective KC depletion. (A)** Scheme of the experimental procedure for mouse genotypes and timeline of ILY and Con A treatments, and the immune cell and chemokine analysis. *ihCD59^+^* and *LysM-Cre^+^ihCD59^+^* mice (n = 3-5 at 4 h, n = 3 at 8 h) were treated with 3 ILY injections (100 ng/g, i.p., at 2 h intervals), followed by Con A injection (12 mg/kg i.v.) 24 h later. **(B)** Number of hepatic infiltrating immune cells at 4 h and 8 h after Con A injection. **(C)** Scatter plot (upper) shows RT2 Profiler PCR Array analysis of cytokine and chemokine gene expression in livers of *ihCD59^+^* (n = 3) and *LysM-Cre^+^ihCD59^+^* mice (n = 3) at 4 h after Con A injection. Fold changes >2.0 and a P-value < 0.05 were considered to be significant variations and are shown in the table. **(D)** LegendplexTM flow assay of proinflammatory chemokines in serum at 4 and 8 h after Con A injection (n = 4-8 mice in each group). Data are shown as mean ± SEM. * P<0.05; ** P<0.01; *** P<0.001, as determined by unpaired two-tailed Student's *t*-test.

In a previous study, we showed that the depletion of myeloid cells (MCs) and KCs ameliorated Con A-induced liver injury by injecting a single dose of ILY 1 h ahead of Con A injection in *LysM-Cre^+^ihCD59^+^* compared with *ihCD59^+^*mice 27,38. However, due to the short time interval between ILY and Con A administrations, the relative contributions of circulating monocytes and KCs remains elusive since they are both depleted during disease onset and progression. To specifically study the role of KCs in Con A-induced hepatitis, we injected Con A at 24 h after one or three ILY injections (Figure 3A), in which KCs are partially and almost completely depleted, respectively (Figure 1C and 1D). Con A injection in *ihCD59*^+^ mice induced severe liver injury as demonstrated by massive liver necrosis (Figure 3B) and the elevation of serum ALT/AST, which was not affected by ILY injection (Figure 3B). However, compared to *ihCD59^+^*mice, one ILY injection partially ameliorated, and three ILY injections completely abolished Con A-induced liver injury in *LysM-Cre^+^ihCD59^+^*mice as demonstrated by 1) less or absence of liver necrosis (Figure 3B) and 2) reduced elevation or normal serum ALT/AST (Figure 3B). Three ILY injections also significantly reduced serum inflammatory cytokines including IL-1α, TNF-α, IFN-γ, MCP-1, IL-12p70, and IL-6 at 4 h and 8 h after Con A injection in *LysM-Cre^+^ihCD59^+^*mice (Figure 3C). Previous studies suggest that these cytokines play critical roles in mediating acute inflammatory responses in Con A-mediated hepatitis 39-44. Another central pathogenic feature of Con A-induced liver injury is the increased infiltration of various immune cells. In the present study, we found that KC depletion by 3 ILY injections in *LysM-Cre^+^ihCD59^+^*mice significantly impaired Con A-induced immune cell infiltration, as demonstrated by decreased number of: 1) monocytes at 4 h and 8 h; 2) CD4^+^ T cell at 4 h; 3) B cells at 8 h; and 4) NK cells at 8 h after Con A injection (Figure 4A and 4B). Taken together, these results indicate that KCs contribute to the pathogenesis of Con A-induced acute liver injury via regulation of inflammatory cytokines and immune cell infiltration.

### KC depletion results in decreased serum protein and reduced hepatitis transcript levels of chemokine CXCL10 in Con A-induced acute hepatitis

A previous study suggested that chemokine networks play a prominent role in immune cell migration during acute liver injury 45. We utilized qPCR array to identify which chemokines are responsible for KC-mediated immune cell infiltration and inflammation during Con A-induced liver injury. At 4 h post Con A injection, we compared the transcript levels of 84 cytokines/chemokines in the livers of *LysM-Cre^+^ihCD59^+^*and *ihCD59^+^*mice pretreated with 3 ILY injections for selective ablation of KCs. qRT-PCR array results showed that 7 chemokines and 9 cytokines in the liver were downregulated in *LysM-Cre^+^ihCD59^+^*mice as early as 4 h after Con A injection (Figure 4C). It is worth noting that IL-1α, IFN-γ, TNF-α and IL-6 transcripts were reduced in livers of *LysM-Cre^+^ihCD59^+^*mice, which is consistent with data in Figure 3C showing their decrease in serum. There was no upregulated cytokine/chemokine transcripts found here, which further confirmed that KC depletion suppressed inflammatory response during liver injury.

We further sought to identify the key chemokine (s) that mainly contribute to the interaction of KCs and infiltrating immune cells during pathogenesis of Con A-induced hepatitis. We examined 13 proinflammatory chemokines in serum via multiplex assay in our established KC cell ablation. Results show that among them, 6 chemokines (CXCL10, CCL2/MCP-1, CCL17, CCL11, CCL20 and CXCL1) were significantly reduced at 4 h or 8 h after Con A injection in ILY-pre-treated *LysM-Cre^+^ihCD59^+^*mice compared to these in control mice (Figure 4D). Interestingly, among these 6 decreased chemokines, CXCL10 is the only one that consistently decreased at both liver transcript level and serum level (Figure 4C and 4D) in liver injury rescued by KC ablation. These results suggest that KC depletion reduced chemokines expression during pathogenesis of Con A-induced acute liver injury. Moreover, CXCL10 may be a crucial mediator between KCs and immune cell immigration.

### Deficiency of CXCL10 ameliorated Con A-induced liver injury

**Figure 5:**
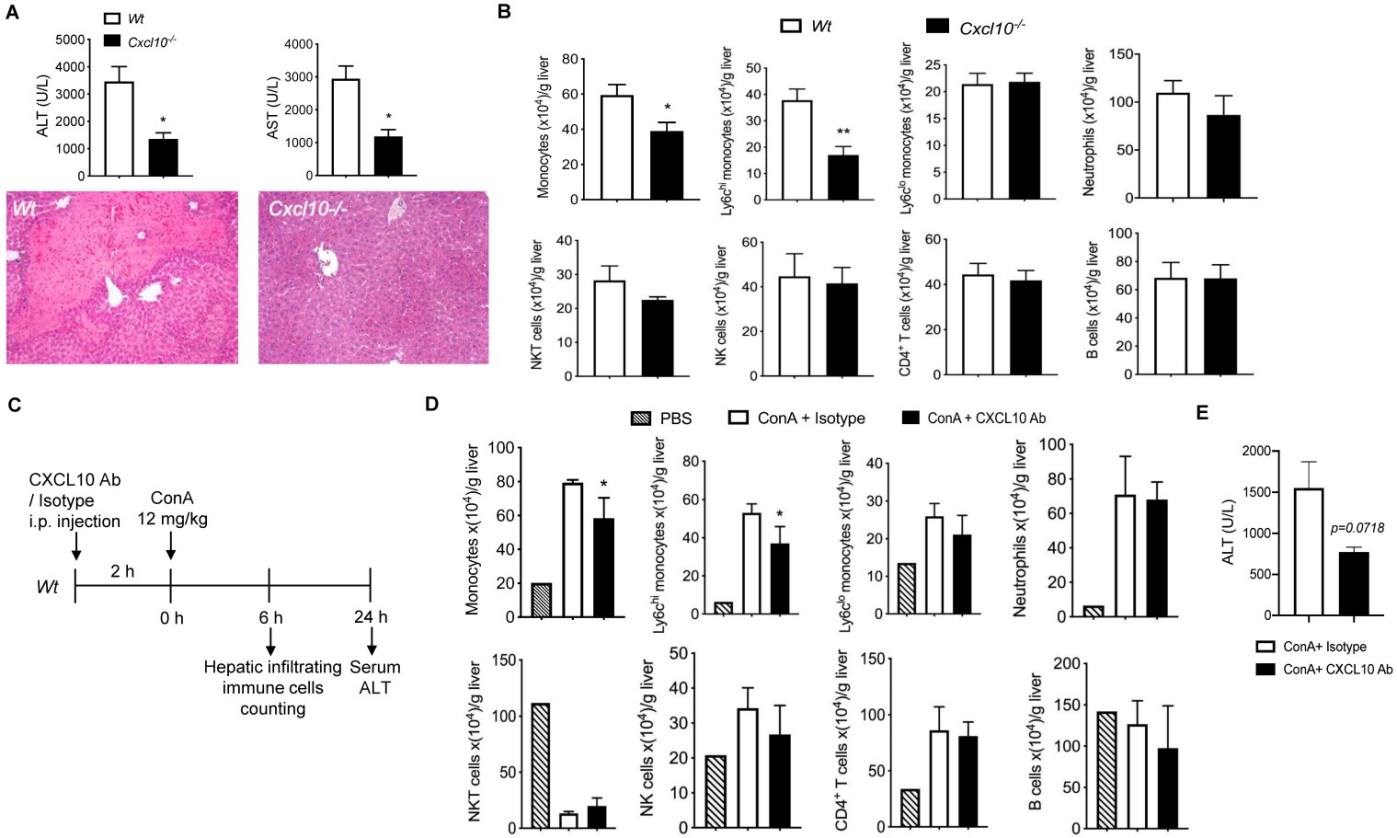
** The deficiency or inhibition of CXCL10 function ameliorated Con A-induced liver injury and inflammatory monocyte infiltration in liver. (A)** H&E staining of liver sections and serum ALT and AST levels in wild-type (Wt, n = 5) and CXCL10 deficient mice (*Cxcl10^-/-^*, n = 4) at 24 h after Con A injection. **(B)** Hepatic infiltrating immune cells at 6 h after Con A injection in *Wt* and *Cxcl10^-/-^* mice.** (C)** Timeline of the CXCL10 neutralizing antibody application (CXCL10 Ab), the Con A treatments, and immune cell analysis. Wt mice were injected CXCL10 Ab or Rat IgG isotype (100 μg per animal) 2 h before Con A injection (12 mg/kg, i.v.) **(D)** Infiltrating immune cell numbers in the livers of CXCL10 Ab or isotype-treated mice at 6 h after Con A injection. (E) Serum ALT level of the mice at 24 h after Con A injection. Data are shown as mean ± SEM. * P<0.05; ** P<0.01, as determined by unpaired two-tailed Student's t-test.

To investigate the role of CXCL10 in acute liver disease, we determined the Con A-induced liver injury in CXCL10 sufficient (*Wt*) and CXCL10 deficient mice (*Cxcl10^-/-^*) 24h after Con A injection. *Cxcl10*^-/-^ mice showed attenuated liver injury compared to Wt mice as demonstrated by 1) less liver necrosis and 2) reduced serum ALT/AST (Figure 5A), suggesting that CXCL10 deficiency could ameliorate acute liver injury. We further demonstrated that CXCL10 deficiency impaired Con A-induced total monocyte infiltration in liver at 6 h after Con A injection, especially the inflammatory Ly-6C^hi^ monocytes, but not alternative Ly-6C^low^ monocytes and other immune cells (Figure 5B). These results indicate that CXCL10-mediated Ly-6C^hi^ monocyte infiltration contributes to Con A-induced liver injury. To further confirm this result, we injected CXCL10 neutralizing antibody (CXCL10 Ab) to block CXCL10 ahead Con A injection in *Wt* mice (Figure 5C). Consistently, we found that CXCL10 antibody specifically decreased total monocytes and Ly-6C^hi^ monocytes in liver injury (Figure 5D). In the present study, CXCL10 antibody displayed a tendency toward decreased ALT level (p=0.0718, shown in Figure 5E) and thus the potential ability to suppress Con A-induced liver injury. Taken together, this is the first study demonstrating that blockade of CXCL10 could reduce Con A-induced inflammatory Ly-6C^hi^ monocyte infiltration in liver and ameliorate Con A-induced liver injury to a certain degree. Consistently, we found that increased CD68 (human monocyte lineage/macrophages marker) and CXCL10 staining in livers of HBV-infection-induced hepatic cirrhosis patients compared with normal liver (Supplementary Figure S7). Of note, almost of CXCL10 signals were co-stained in CD68 signals, further indicating that in liver, CXCL10 is produced by CD68 positive cells such as KCs and macrophages. This finding further supports the critical role of CXCL10 in hepatitis B and C virus-induced liver disease and human nonalcoholic steatohepatitis (NASH) 15,22,46,47.

### KC depletion increased the permeability of liver sinusoidal endothelial cells

**Figure 6:**
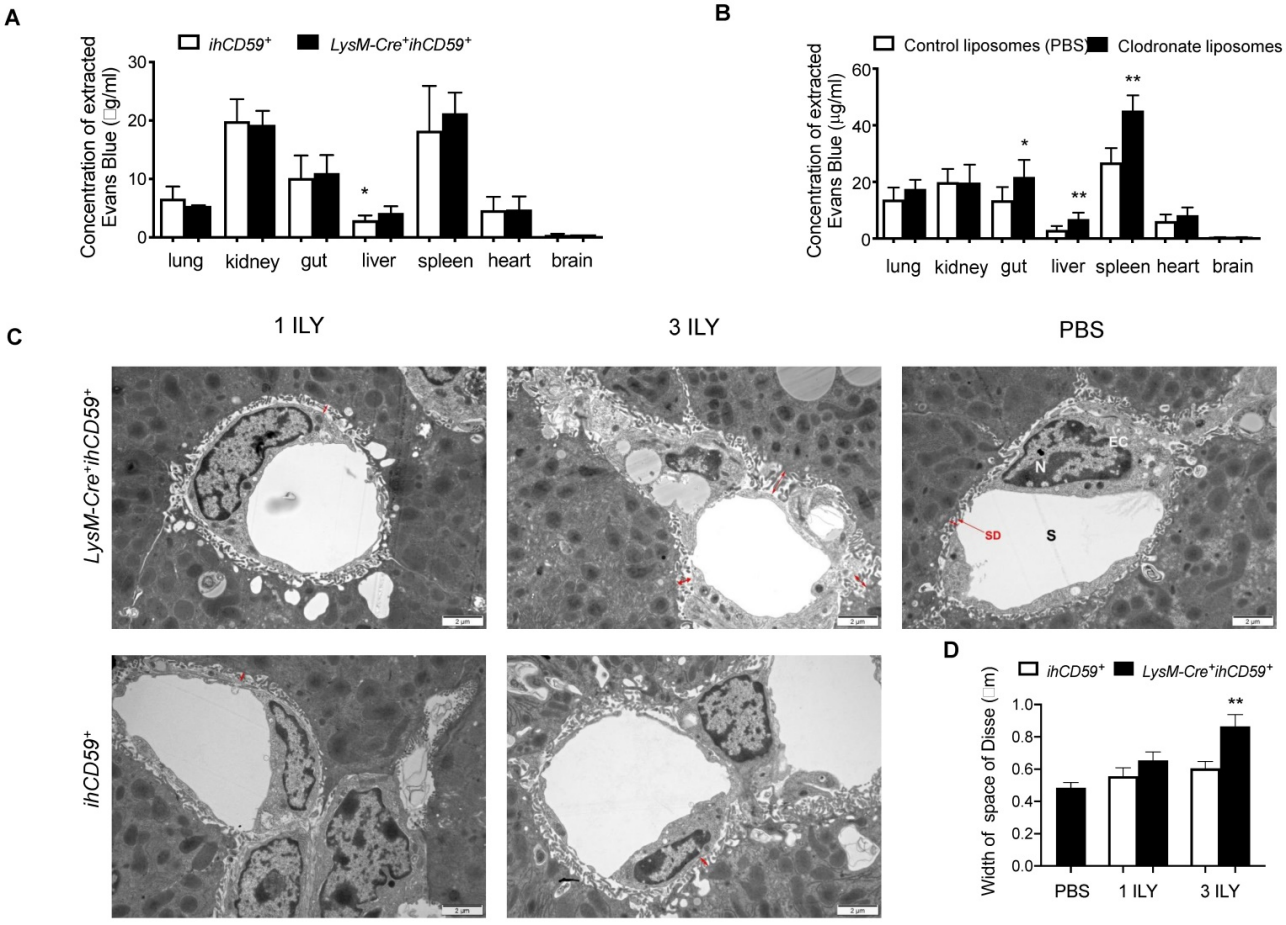
** KC depletion increased the permeability of liver sinusoidal vascular wall. (A)** Concentration of Evans blue extracted from different tissues of *ihCD59^+^* and *LysM-Cre^+^ihCD59^+^* mice (n=3-6) at 24 h post 3 ILY injections (100 ng/g, i.p.). **(B)** Evans blue concentration in different tissues at 24 h after Wt mice (n = 4 - 6) received clodronate liposomes or control liposomes (PBS) injection (10 μl/g body weight, i.v.). **(C, D)** Representative transmission electron microscopy (TEM) of space of Disse (**C**, red arrows) and liver sinusoidal endothelial cells and the quantification of width of space of Disse (D) in *ihCD59^+^* and *LysM-Cre^+i^hCD59^+^* mice (n=2 in each group) treated with 1 or 3 ILY injections. S, sinusoide; SD, space of Disse; EC, endothelial cell; N, nuclear. Data are shown as mean ± SEM. * P<0.05; ** P<0.01; *** P<0.001, as determined by unpaired two-tailed Student's t-test.

KCs and liver sinusoidal endothelial cells (LSECs) are major components of the hepatic sinusoid. Physiologically, LSECs represent a permeable barrier between blood and liver parenchyma, restricting the diffusion of solutes and filtration of immune cells from blood 48. As for KCs, they are adherent to the sinusoidal endothelial layer, where they uniquely capture signals from blood and contribute to the maintenance of hepatic immune stability. To determine the effect of KC depletion on liver vascular permeability, we examined the diffusion of Evans blue (EB) dye from peripheral to different tissues after KC depletion in *LysM-Cre^+^ihCD59^+^*mice. We found that after portal vein perfusion, liver is the only tissue showing significantly increased EB deposition among all tissues examined in *LysM-Cre^+^ihCD59^+^*mice compared to *ihCD59^+^*mice (Figure 6A), suggesting that KC depletion increased the permeability of hepatic vascular walls in physiological condition. Interestingly, by using chlodronate liposomes to eliminate macrophages, we also found increased EB depositions not only in liver but also in gut and spleen of *Wt* mice at 24 h after liposomal chlodronate injection (Figure 6B). We further examined the ultra-structure of sinusoid by transmission electron microscope (TEM). TEM observation revealed that the space of Disse between hepatocytes and LSECs was significantly increased in 3 ILY-treated but not 1 ILY-treated *LysM-Cre^+^ihCD59^+^*mice compared with *ihCD59^+^* mice (Figure 6C and 6D), suggesting that extensive KC depletion resulted in an increase of sinusoidal wall permeability. Importantly we did not see impairment of LSEC structure in our study (Figure 6C). Taken together, these results demonstrated that efficient KC depletion increased the sinusoidal vascular permeability and induced edema of the sinusoid.

## Discussion

Here, we documented that the elimination of KCs suppresses immune reactions in liver by reducing the number of infiltrating immune cells and downregulating a number of cytokines and chemokines. Using our previously established rapid ILY/hCD59 cell ablation model 27,28, we successfully developed a selective and transient KC ablation method to dynamically and comprehensively investigate the function of KCs in static condition. Since KCs and infiltrating monocytes are two main populations consisting of hepatic macrophages 7, it has been a challenge for us to differentiate these two populations in studies under either physiological or pathological conditions. Although a variety of monocyte and KC ablation approaches (clodronate liposomes, chloride, diphtheria toxin/diphtheria toxin receptor (DT/DTR), and gadoliniumhave been used to dissect the function of KCs in liver diseases 3,23,24, they have not been used to dynamically and comprehensively investigate the function of KCs in static condition. These methods could not clearly differentiate KC-associated immune and non-immune responses from KC loss phase to regeneration phase due to the long delay (days) in inducing cell death (days) by initiating apoptosis 25. They have undesirable side effects, including inhibition of KC function 26. In contrast, we created our ILY/hCD59 cell ablation model based on findings that intermedilysin (ILY), exclusively lyses human cells but not cells from any other species through binding to the human complement regulatory protein CD59 (hCD59) 30. ILY is a cholesterol-dependent cytolysin that is secreted by *Streptococcus intermedius*. ILY specifically lyses human cells by binding to hCD59 and forming a pore 27,28,30. One unique feature of our ILY/hCD59 cell ablation model is the rapidity with which ILY mediates necrotic effect on hCD59 expressing cells: within seconds in vitro and in few minutes in vivo 27,28. This means we are able to ablate monocytes and KCs in a very short period. Interestingly, we found that the numbers of monocytes and neutrophils in peripheral blood and KCs in the liver were recovered within short periods after 1 ILY treatment (3-5 h for monocytes and neutrophils, and 12 h for KCs as shown in Figure 1A, 1B and 1C). The underlying mechanism for the fast recovery remains unknown and requires further investigation. Previously, we documented that ILY-mediated peripheral hCD59 expressing cell ablation occurs within 10 min 27. It is possible that one dose of ILY is insufficient to ablate the monocytes resided in bone marrow and tissues, and KCs in liver. Thus, the fast recovery of circulating monocytes and neutrophils, and hepatic KCs may result from the release of monocytes from bone marrow and tissues 49-51, and the KCs regeneration derived from KCs proliferation or infiltrating monocytes 52, respectively. Further, based on our finding that circulating monocytes returned to the baseline level at 24 h after initial ILY injection (Supplementary Figure S3) while KCs still maintained at the lowest level at this time (Figure 1D), we picked 24 h post 3 ILY injections as the best time point for selective and transient KC ablation and further functional studies. With this model, we have a chance to reveal the dynamic changes of immune responses after KC depletion. We found loss of KCs induced a significant decrease in hepatic infiltrating immune cells (monocytes, CD4^+^ T cells, CD8^+^ T cells and B cells) at 14 and 24 h post ILY injection, and continuous downregulation of cytokine/chemokine transcriptional levels (IL-12β, CXCL13, CXCL9 and CCL5) in livers at 2, 14 and 24 h post ILY treatments in steady state. These results indicate that KCs play very important roles in maintaining hepatic homeostasis through modulating immune cell infiltration and immune response. However, it remains unclear how these cytokines and chemokines contributes to maintaining immune cells and the immune response.

In pathological conditions, KC activation is an essential response of liver to infection or injury: The ensuing inflammatory response protects from infection and limits cellular and organ damage to the host organism 53. Thus, in many conditions, KCs play an important protective and healing role. However, in some types of insults to liver, KCs could not appropriately control or resolve its state of activation, which contributes to a number of inflammatory diseases in the liver. Due to the dual protective and harmful role of KCs, study of KC function should be critically timed during the dynamic stages of liver injury 54. In our study, the elimination of KCs completely protects against Con A-induced hepatitis by decreasing serum inflammatory cytokines such as IL-1α, TNF-α, IFN-γ, MCP-1, IL-12, and IL-6, relevant to Con A-induced hepatitis 39-42. The pathogenic roles of IL-1α, TNF-α, IFN-γ, and IL-6 in Con A-induced hepatitis have been extensively established in previous studies 39-42. Research on MCP-1 and IL-12 functions in Con A-induced liver injury is still contradictory. Previously, their pathogenic roles have been documented using MCP-1 receptor deficient mice 44, with IL-12 neutralizing antibody and exogenous IL-12 43. However, anti-pathogenic roles of MCP-1 and IL-12 in Con A-induced hepatitis have been demonstrated by using MCP-1 neutralization and IL-12 knockout approach 41,55, respectively. Those contradictory results may result from the application of different approaches and mouse models. Nevertheless, both MCP-1 (a monocyte and T lymphocyte chemoattractant) and IL-12 (a heterodimeric cytokine produced by macrophages and dendritic cells) have a central role in cell-mediated immune responses and have been linked to the progression of autoimmune diseases such as arthritis and multiple sclerosis 41,43,56,57. These results support our findings that KCs also contribute to maintaining immune responses in the pathological condition.

Another finding in our study is that KCs play critical role in maintaining infiltrated immune cells such as monocytes, T cells, B cells, and NK cells in pathological conditions, which has been recognized by the previous studies 3,7. The underlying mechanism might be the decrease in a list of chemokines (Figure 4C and D) after KC ablation in Con A-induced hepatitis. Among the reduced chemokines, we have identified CXCL10 as a key chemokine to participate in recruiting inflammatory monocytes during Con A-induced hepatitis. Previous clinical studies have also indicated that CXCL10 may play a pathogenic role in NASH and hepatitis C virus (HCV)-induced liver disease 15,22,58. We also found an increased CXCL10 staining in HBV infection-induced hepatic cirrhosis but not in normal liver. This finding further supports the critical role of CXCL10 in hepatitis virus-induced liver disease 15, 22, 46. In addition, CXCL10 has been found in hepatocytes surrounded by infiltrative mononuclear cells in chronic hepatitis. It is a specific chemoattractant for T lymphocytes in the inflammatory liver tissues and brain tissues 59,60. In contrast, our studies reported here show that both inhibiting CXCL10 activity and knocking out CXCL10 function specifically reduce the infiltrating inflammatory monocytes, thereby leading to attenuating Con A-induced hepatitis. Previous studies also suggest that knocking out CXCL10 function is protected against diet-induced NASH in an obesity-independent manner 61. Macrophage-associated inflammation appears to be the key player in the CXCL10-mediated sterile inflammatory response in murine NASH 47,61,62. Targeting CXCL10 has also been shown to have a novel therapeutic means to ameliorate liver IRI in clinical setting 63. Those results indicate that CXCL10 produced in the liver plays an essential role in the pathogenesis of acute and chronic liver diseases through the recruitment of infiltrating monocytes. Anti-CXCL10 may have a therapeutic potential for treatment of these liver diseases. Of note, although the pathogenic role of CXCL10 and the induction or production of CXCL10 associated with KC activation has been documented in acute liver disease, the upstream and downstream signaling pathways of CXCL10 for KC activation-mediated CXCL10 production remain elusive. In addition, the pathogenic role of other chemokines such as CCL2 (MCP-1), CCL17, CCL11, CCL20 and CXCL1 in liver diseases remains unclear and requires further investigation. Further, a previous study shows that KCs are important in rapidly producing cytokines and chemokines, including IL-1β, TNF-α, IL-4, IL-6, IL-10, MCP-1 and CCL5 64. Some of these molecules such as MCP-1, TNF-α, and IL-6 were also found decreased after KC ablation in our study. Thus, the reduction of cytokines/chemokines observed in the current study may be directly due to KC depletion, or indirectly to KC loss-mediated impairment of their production in other cell types.

Finally, our results reveal that the depletion of KCs resulted in increased vascular permeability, which indicates that KCs interact with liver sinusoid endothelial cells to maintain the integrity of hepatic vasculature. Unlike vascular endothelial cells (ECs), LSECs do not have an organized basement membrane and are fenestrated, which allows nutrients in the parenchyma, and crosstalk with hepatocytes 65. KCs are overlaid on top of LSECs in the sinusoidal lumen side. A previous study suggested that during septic inflammation, KCs are involved in protecting LSECs and, indirectly, hepatocytes from further injury 66. Interestingly, another study shows a critical role for macrophages in the regulation of mesenteric vascular barrier function in the steady condition 67. Our finding advances our understanding of KCs' interplay with LSECs for maintaining hepatic microcirculation, although the underlying molecular mechanisms remain unclear. These studies directly highlight the importance of tissue resident macrophages in maintaining vessel barrier integrity and controlling vascular permeability, which warrants further investigation.

## Conclusions

By using our rapid ILY/ihCD59 cell ablation mice model, we unravel the key role of KCs in networking with other cells to maintain hepatic immune homeostasis in both steady and pathological conditions. The loss of KCs directly causes reduction of infiltrating immune cells and decrease of cytokine/chemokine expression in the liver. The increased permeability of the sinusoidal wall after KC depletion indicates that KCs participate in regulation of hepatic vascular function and maintenance its integrity in steady state. Furthermore, our data suggest the efficiency, rapidity, and small usage of endotoxin-free ILY make ILY/ihCD59 cell ablation mice a suitable animal model for further timed and dynamic investigations of immune cell functions in both physiological and pathological conditions.

## Supplementary Material

Supplementary figures and tables.

## Abbreviations

KCs: Kupffer cells
LSECs: sinusoidal endothelial cells
Con A: concanavalin A
HSCs: hepatic stellate cells
DT: Diphtheria toxin
DTR: Diphtheria toxin receptor
*ihCD59^+^*: Cre-inducible hCD59 transgenic mice
qRT-PCR: Quantitative Reverse Transcription Polymerase Chain Reaction
AST: Aspartate transaminase
ALT: Alanine Aminotransferase
and NASH: nonalcoholic steatohepatitis
