# Supplementary material for: Kupffer cells promote T-cell hepatitis by producing CXCL10 and limiting liver sinusoidal endothelial cell permeability
Source: Theranostics. 2020 Jun 1;10(16):7163–77. doi: 10.7150/thno.44960 (PMC7330839; doi:10.7150/thno.44960)
Supplement: Supplementary file 1 — Supplementary figures and tables. [file thnov10p7163s1.pdf]

Supplemental Figure 1

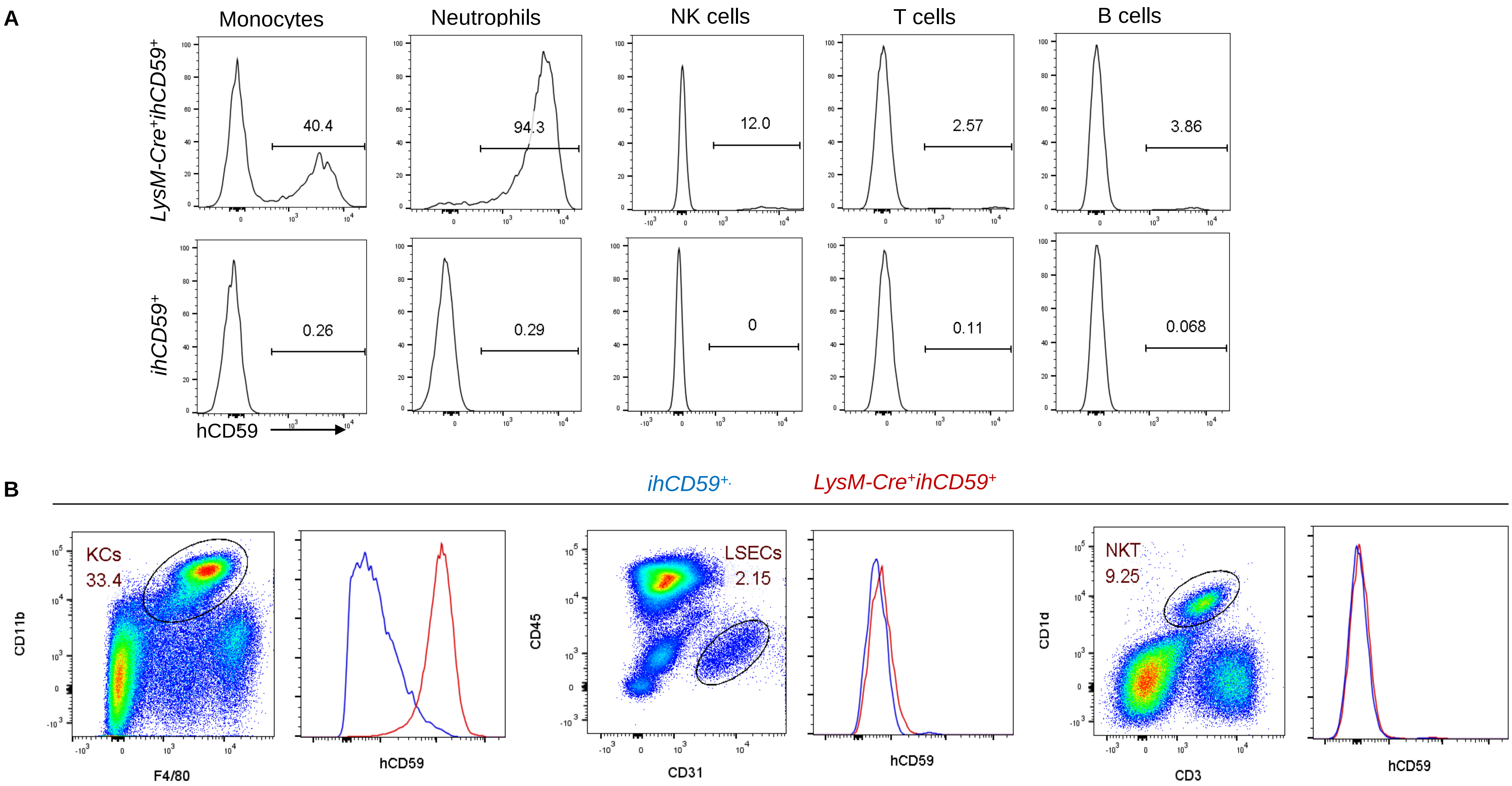

Supplemental Figure 1. hCD59 expression on *LysM-Cre⁺ihCD59⁺* mice. (A) Circulating and (B) hepatic immune cells of *LysM-Cre⁺ihCD59⁺* mice were isolated and examined for hCD59 by flow cytometry.

Supplemental Figure 2

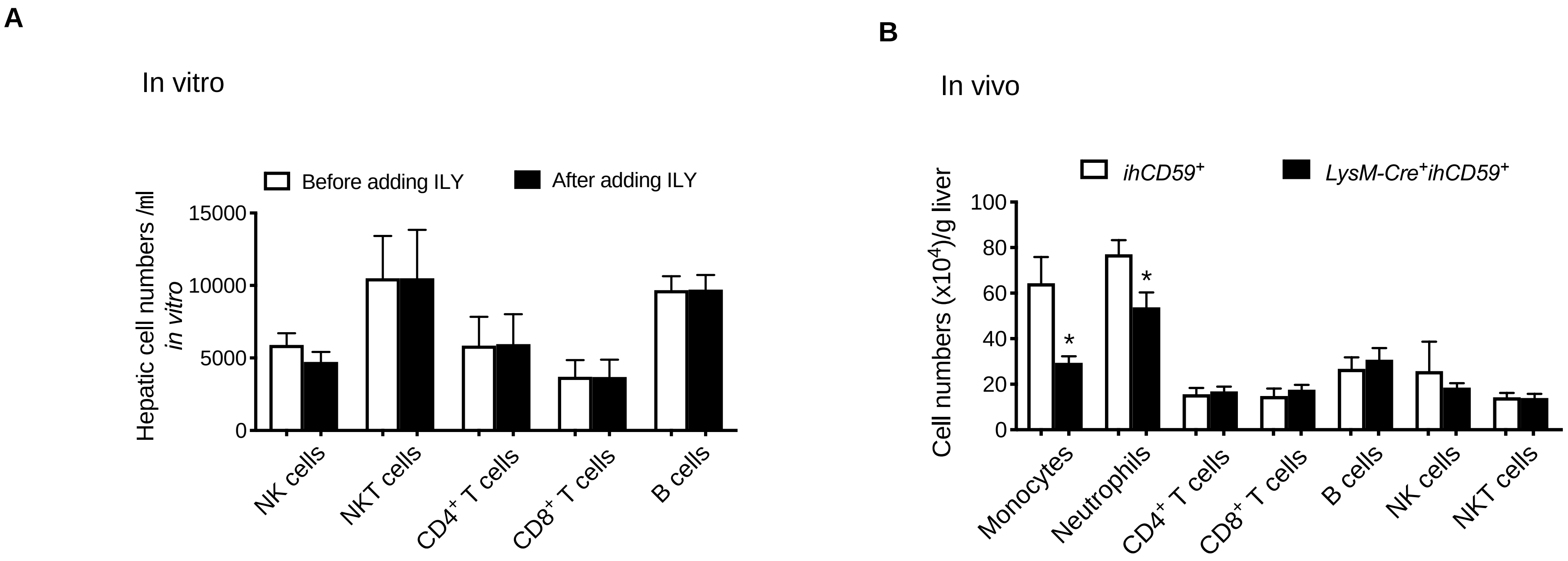

Supplemental Figure 2. No off-target effects of ILY both in vitro and in vivo. (A) Hepatic immune cells isolated from *LysM-Cre*$^{+}$*ihCD59*$^{+}$ mice (n=4) were counted by flow cytometry before and after exposure to ILY in vitro. The ILY dose is 1.2 μg/ml, 350 folds of ILY IC50, which is high enough to complete lyse hCD59 expressing cells. Then cells were incubated at 37℃ for 30 min. **(B)** *LysM-Cre*$^{+}$*ihCD59*$^{+}$ mice (n=4) were injected with 1 ILY (100 ng/g, i.p.). Hepatic immune cells were analyzed 2 h post the injection.

Supplemental Figure 3

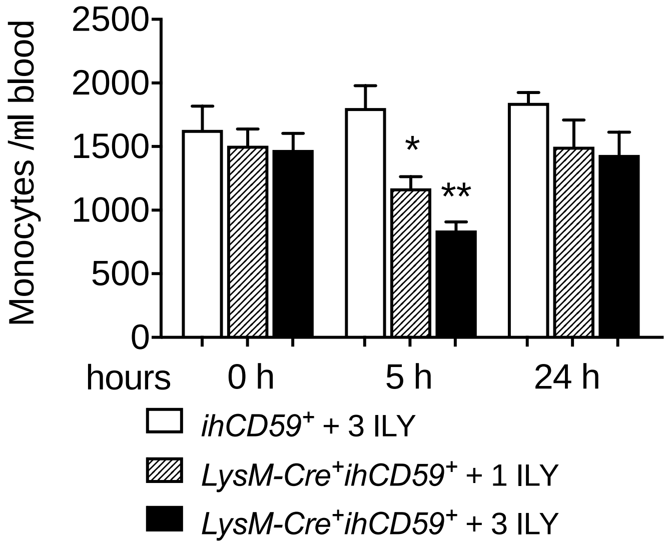

Supplemental Figure 3. Dynamic changes in circulating monocytes after the injection of ILY with two different dosing regimens to *LysM-Cre⁺ihCD59⁺* mice show dose dependent effect. *LysM-Cre⁺ihCD59⁺* mice (n=4) were injected either 1 ILY (150 ng/g, i.p.) or 3 ILY (100 ng/g, i.p. at 2-hour intervals). ihCD59⁺ mice received 3 ILY injections (100 ng/g, i.p. at 2-hour intervals) was used as control (n=4). Number of circulating monocytes were analyzed before (0 h) and 5 h, 24 h post injections.

Supplemental Figure 4

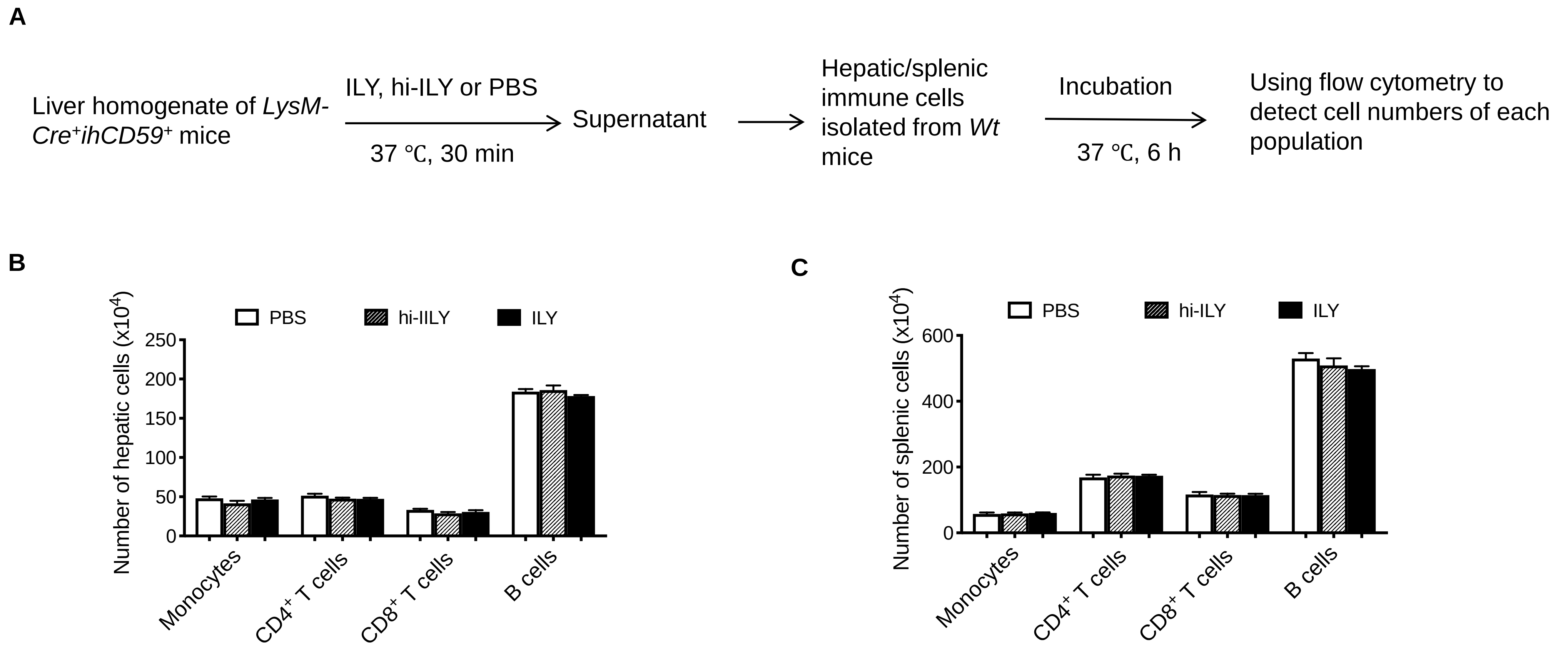

Supplemental Figure 4. **Cellular contents released from ILY-lysed cells are not toxic to other immune cells**. **(A)** Timeline of the experimental procedure. ILY (0.6 μg/ml, 174 folds of ILY IC50, which is high enough to completely lyse hCD59+ cells), heat-inactive ILY (hi-ILY, 0.6 μg/ml) and PBS were respectively added in liver homogenate of *LysM-Cre^{+}ihCD59^{+}*mice (n=4). After incubation at 37 ℃ for 30 min, the supernatants were collected and respectively incubated with equal amount of WT hepatic or splenic immune cells at 37 ℃ for another 6 h. Then the number of hepatic **(B)** or splenic **(C)** immune cell populations incubated with ILY-treated, hi-ILY-treated and PBS-treated supernatants were analyzed by flow cytometry (n=3).

Supplemental Figure 5

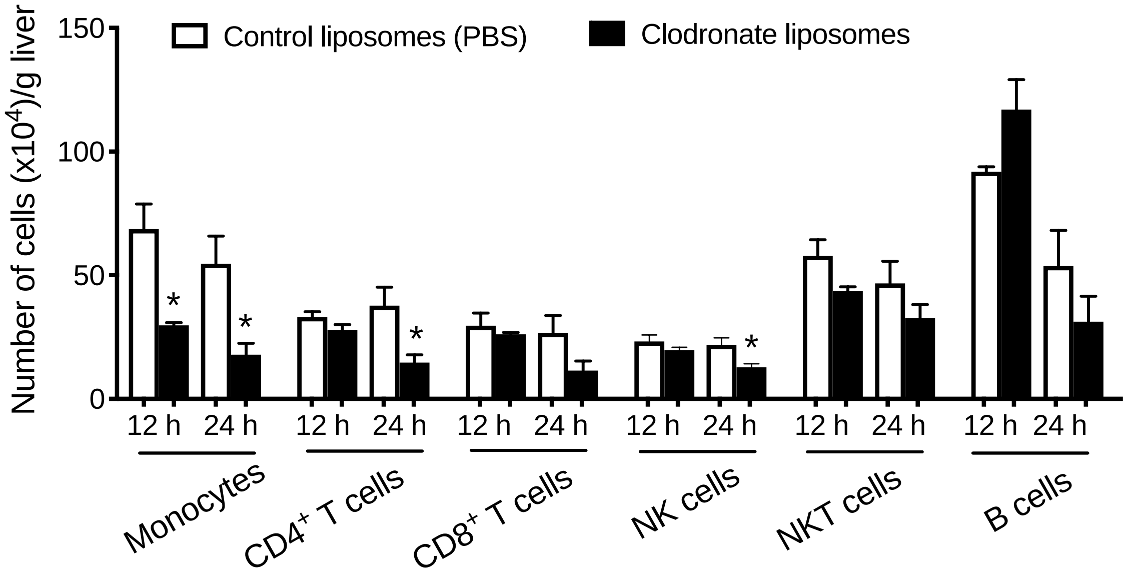

Supplemental Figure 5. KCs ablated by Clodronate liposomes induced reduction in the hepatic infiltrating monocytes, CD4$^+$T and NK cells. *Wt* mice (n=3 for 12 h and n=6 for 24 h) were injected with clodronate liposomes or control liposomes (PBS) (10 ul /g body weight, i.v.). Hepatic immune cells were analyzed 12 h and 24 h post injection by flow cytometry.

Supplemental Figure 6

A

2 h after 1 ILY injection

Gene Symbol	Fold Regulation
Cxcl3	10.09
Csf3	7.26
Il3	4.07
Il22	3.89
Cxcl1	3.56
Il11	3.39
Il9	3.25
Il24	2.84
Il17a	2.83
Ccl17	2.78
Il4	2.70
Tnfsf11	2.64
Il12a	2.54
Osm	2.53
Ccl11	2.46
Il17f	2.41
Mstn	2.35
Ppbp	2.23
Il21	2.20
Cd70	2.19
Il5	2.14
Il13	2.11
Lta	2.04
Il1rn	2.03
Nodal	2.01

B

14 h after 3 ILY injections

Gene Symbol	Fold Regulation
Spp1	5.28

C

24 h after 3 ILY injections

Gene Symbol	Fold Regulation
Spp1	9.48
Csf3	8.37
Adipoq	7.26
Cxcl5	4.69
Ccl7	4.45
Ccl12	4.30
Il13	3.89
Pf4	3.61
MGDC	3.15
Il12a	3.14
Il21	3.11
Cd70	2.96
Hc	2.82
Il3	2.73
Ccl2	2.67
Bmp4	2.56
Cxcl11	2.50
Ccl19	2.46
Il22	2.29
Fasl	2.28
Lta	2.27
Il9	2.21
PPC	2.18
Il24	2.14
RTC	2.14
RTC	2.14
PPC	2.14
RTC	2.14
PPC	2.11
Osm	2.09
Thpo	2.08

Supplemental Figure 6. Lists of increased cytokine/chemokine gene transcripts in the liver of *LysM-Cre$^+$ihCD59$^+$* mice after KCs ablation.

Supplemental Figure 7

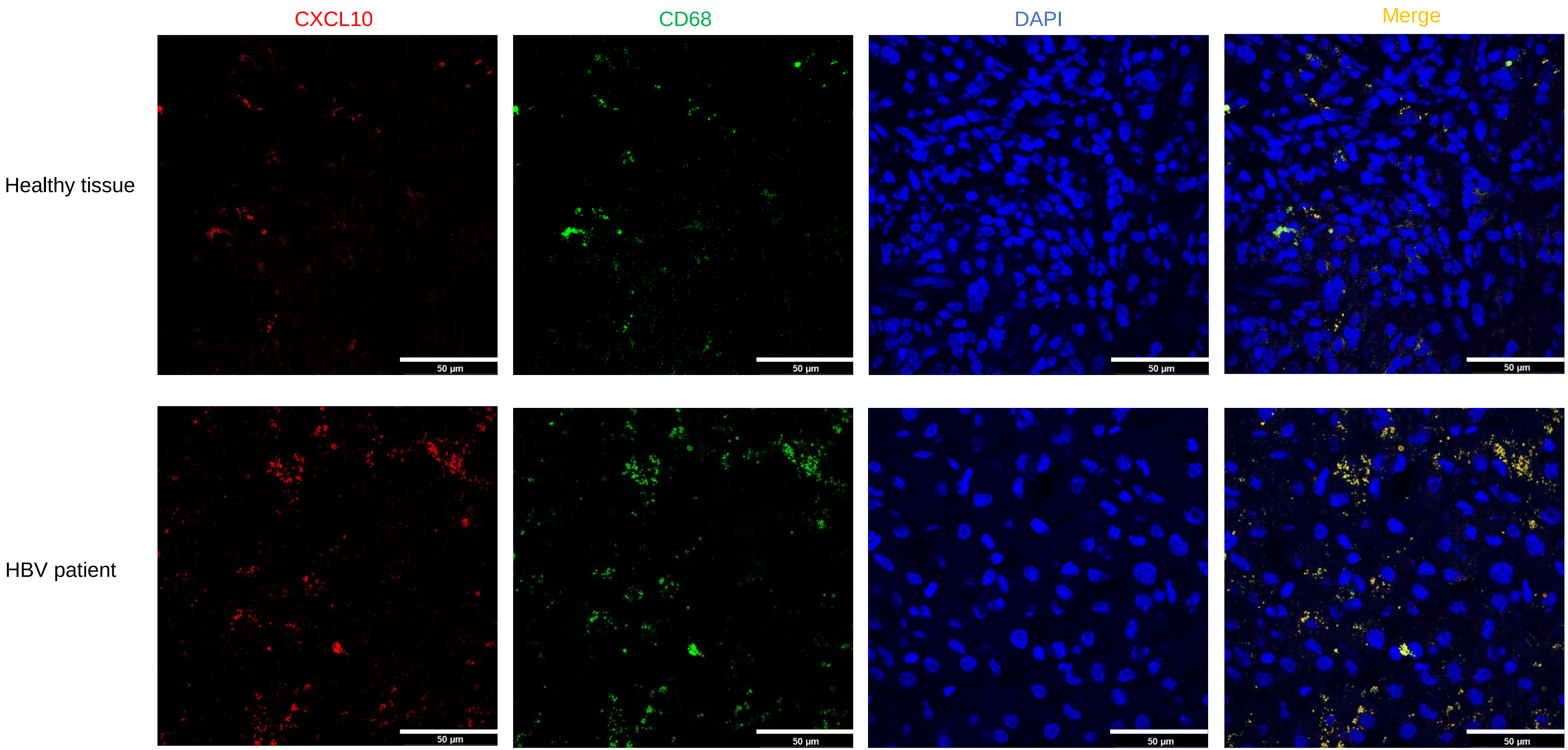

Supplemental Figure 7. Immunofluorescent staining of CD68 and CXCL10 in livers biopsy from healthy human individuals and HBV-induced cirrhosis patients. Representative Immunofluorescence image of sections from a HBV-induced cirrhosis patient's liver biopsy stained with DAPI (blue), anti-CD68 mAb (green) and CXCL10 antibody (red). Bar=50 µm.

Supplemental Figure 8

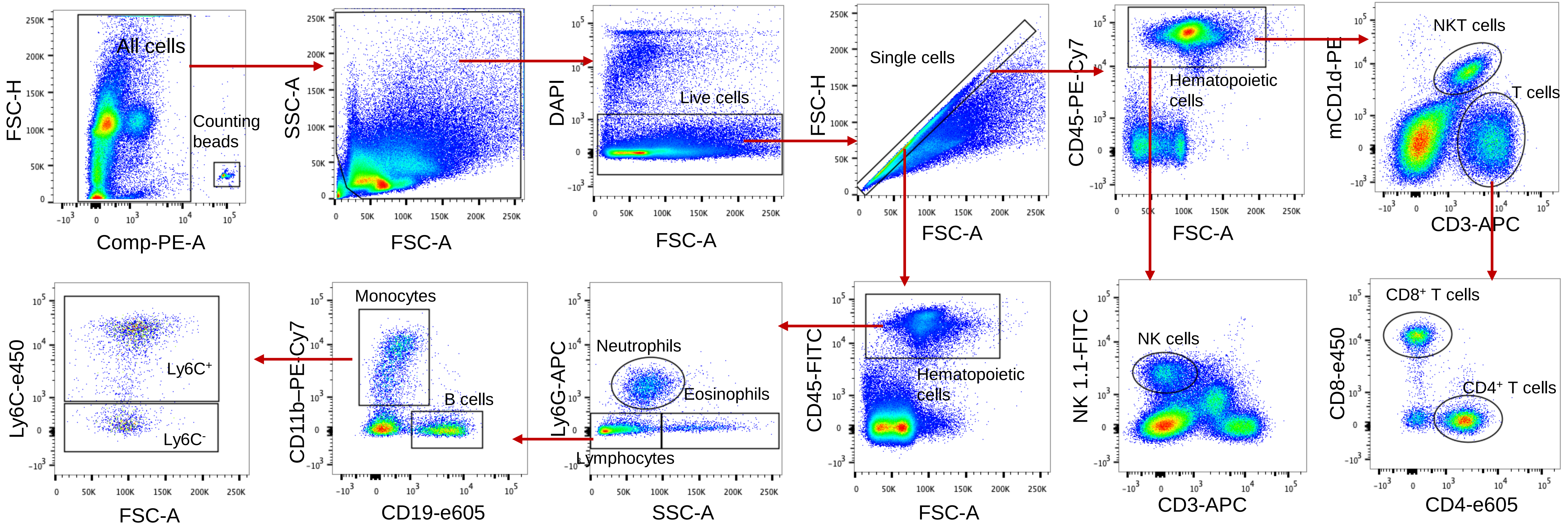

Supplemental Figure 8. Flow cytometry analysis gating strategy of hepatic immune cells in FlowJo.

Supplemental Table 1.

List of antibodies used in flow cytometry

Antibody	Clone	Cat Number	Company
CD45	30-F11	48-0451-82	eBioscience
CD45	30-F11	11-0451-82	eBioscience
CD11b	BM8	17-4801-80	eBioscience
CD3	17A2	17-0032-82	eBioscience
CD4	RM4-5	83-0042-42	eBioscience
CD8	53-6.7	48-0081-82	eBioscience
CD19	1D3	83-0193-42	eBioscience
NK1.1	PK136	11-5941-81	eBioscience
F4/80	BM8	123133	Biolegend
hCD59	OV9A2	12-0596-42	eBioscience
Ly6C	HK1.4	48-5932-82	eBioscience
Ly6G	1A8	127613	Biolegend
Purified CD16/32	93	14-0161-85	eBioscience
CD16/32(FcγRII/III)	93	48-0161-80	ebioscience
CD31	390	11-0311-82	ebioscience
mCD1d-PBS57-PE-tetramer			NIH Tetramer Core Facility
